# Novel monoclonal antibodies against *Plasmodium falciparum* histidine-rich protein 2: development and application in rapid diagnostic tests of malaria in hyperendemic regions of China and Myanmar

**DOI:** 10.1186/s12866-015-0429-1

**Published:** 2015-05-12

**Authors:** Keren Kang, Emmanuel E Dzakah, Wenmei Li, Mingquan Xie, Xiaochun Luo, Hui Liu

**Affiliations:** School of Bioscience and Bioengineering, South China University of Technology, Guangzhou, 510006 China; National Engineering Laboratory of Point-of-Care Tests, Guangzhou Wondfo Biotech Co. Ltd, Guangzhou, 510663 China; Yunnan Provincial Center for Parasitic Diseases Control and Prevention (NIPD), Yunnan, 650000 China; Department of Molecular Biology and Biotechnology, School of Biological Sciences, University of Cape Coast, Cape Coast, Ghana

**Keywords:** Malaria, Histidine-rich protein 2 exon II, Monoclonal antibodies, Point-of-care test, Immunochromatographic test

## Abstract

**Background:**

Malaria presents a considerable threat to public health. Histidine-rich protein 2 (HRP 2) is the major protein released into human blood upon infection by *Plasmodium falciparum.* In this study, we aimed to evaluate the immunogenicity of HRP 2 exon II and the efficacy of novel monoclonal antibodies (mAbs) against HRP 2 for Point-of-Care Test (POCT).

**Methods:**

The recombinant protein was expressed in soluble form in *E. coli* and used to immunize mice for mAb production. Two IgG1 mAbs (1A5 and 1C10) with high affinity, specificity and sensitivity for both native and recombinant HRP 2 were selected after fusion of mouse spleen with myeloma cells. The affinity constant of 1A5 and 1C10 were 7.15 and 4.91 × 10-7 L/mol, respectively. Subsequently, an immunochromatograhic assay was used for screening of clinical samples in endemic regions of China and Myanmar.

**Results:**

The immunochromatographic test retrospectively showed an overall sensitivity of 99.07%, and specificity of 100%. Sensitivity at parasite densities < 200, 200–2000, and > 2000 parasites/μL was 87.5, 98.7, and 100%, respectively.

**Conclusions:**

These results suggest that HRP 2 exon II contains immunogenic sites similar to those of the native antigen and can be used for the development of mAbs suitable for malaria diagnosis in endemic communities.

## Background

Malaria, a disease caused by *Plasmodium species*, is one of the oldest and largest health challenges affecting 40% of the world population [1]. There were an estimated 627, 000 malaria deaths in 2012, including 91% in Africa, mainly caused by *P. falciparum* infection, the most lethal malarial plasmodia responsible for the cerebral form of the disease. Approximately 86% malaria deaths are children under 5 years of age [2-4]. These estimates rank malaria as one of the top three killers among infectious diseases in the world. Although *Plasmodium falciparum* prevalence rates in most parts of China and Myanmar have been brought under control, very high transmission rates still remain in certain regional communities [5]. Due to the economic boom in China, cross border transmission has resulted in increasing malaria incidence in recent years. Current economic globalization trends coupled with marked movement of people have accelerated the incidence of *Plasmodium* related cases with increased antimalarial drug resistance in Southeast Asia, including China [5]. The Yunnan Province alone counts over ten million cases of malaria among border and immigration officers, an indication of the prevalence of malaria at both sides of the border. Continuous migration of the population in border areas makes it extremely difficult to implement malaria control programs. Available data in recent years have also shown imported cases of malaria in Henan, Hebei, Fujian, Chongqing, Shanghai, Jiangxi and among others [6].

Inaccurate and ineffective diagnosis of *P. falciparum* in these regions has resulted in drug resistant species, pointing to the need for improved diagnosis and monitoring of the disease. The total eradication of malaria is one of the urgent aims of the United Nations Millennium Development Goals. The methods recommended by WHO for *P. falciparum* diagnosis include microscopic examination, immunological tests, and PCR methods [7-9].

Since the launch of WHO’s *T3* initiative in 2012, all malaria-endemic and donor countries should ensure that every suspected case of malaria is tested and treated, [10] requiring increased developent of rapid diagnostic tests (RDTs). Hence the use of fast, accurate, and easy on-site detection methods and reagents as monitoring medical tools for early diagnosis and treatment of malaria in regions of high transmission and prevalence is particularly important. Several detection antibodies against different *Plasmodium* antigens have been described, of which *Plasmodium falciparum* specific histidine-rich protein 2 (HRP 2), and *Plasmodium* lactate dehydrogenase (pLDH) and aldolase are common to all four *Plasmodium* species [11]. The PfHRP 2 gene is located on chromosome 8 of the parasite and comprised of both exons I and II, encoding a 309-amino acid protein. Sequence variations among the different strains range between 800 to 1300 base pairs. The PfHRP exon II alone encodes 287 amino acids composed of 34.5% histidine and 35 repeats of the tripeptide His-His-Ala sequences. HRP 2 is released upon rupture of parasitized erythrocytes at late-stage [12] and is capable of reversing the tightly balanced activities of anticoagulant factors that maintain homeostasis [13].

The most convenient technique for clinical diagnosis of malaria is the use of rapid diagnostic test kits, which depends on the use of mAbs against the HRP 2 antigen. These tests are particularly important since they can be used in field diagnostics (point-of-care test, POCT) to screen large populations without the requirement of trained laboratory personnel or equipment. Several tests targeting HRP 2 are available, with various specificities, sensitivities, and temperature tolerances, illustrating the difficulties and challenges facing current RDTs [14]. The difficulties associated with RDTs include genetic variability in the HRP 2 gene and the persistence of antigens in the bloodstream following the elimination of parasites [14]. For instance, ParaSight F, a sensitive, specific, simple, and fast dipstick assay, uses mAb IgG1, a subclass of IgG [15,16]. However, HRP 2-based RDTs have given false-positive results and there has been some debate as to the cause of these false positives [14]. Therefore, the development and production of mAbs with high affinity, specificity and sensitivity will help facilitate its use as POCT.

In this study, we aimed to express the HRP 2 exon II antigen retaining the essential epitopes that can serve as immunogens for use in developing mAbs against HRP 2 for POCT immunochromatographic assay, according to guiding principles formulated by the WHO [10]. The results of assays were compared with those of microscopic examination known as gold standard for clinically screening of malaria samples in endemic regions of China and Myanmar.

## Methods

### Sample collection and reference samples

**Figure 1:**
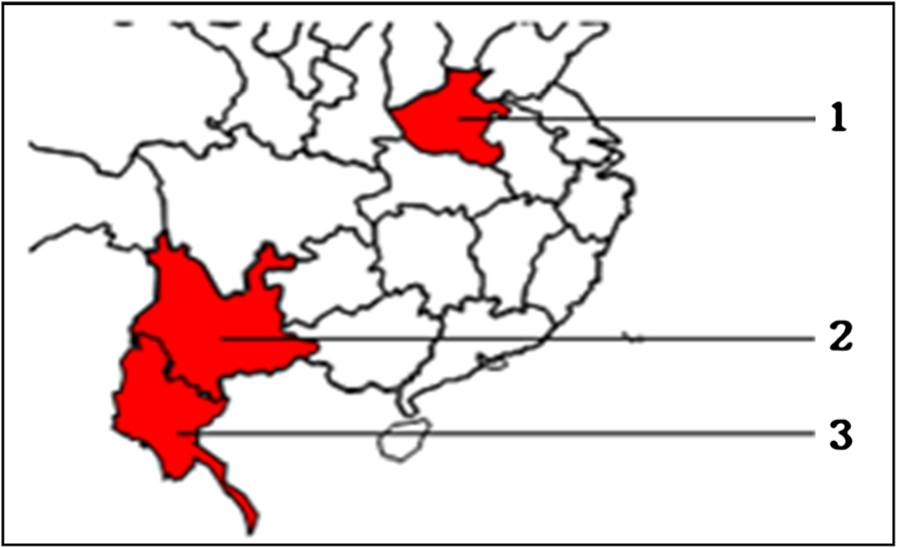
Geographical distribution of malaria endemic areas in China and Myanmar. 1, Henan Province (n=90); 2, Yunnan Province (n=202); 3, Myanmar (n=224).

Clinical samples (n=516) were collected between 2008 and 2012 from patients (6–50 years old) with fever and suspectable for malaria from Henan (n=90) and Yunnan (n=202) provinces of China (total n=292) and from Myanmar (Burma) region (n=224), where malaria is endemic and is caused mainly by *Plasmodium falciparum* as shown in Figure 1. The study was approved by the ethics committee of Yunnan Institute of Parasitic Diseases. All participants or legal guardians signed informed consent before participant enrolment and sample collection. Complete anonymity was maintained at each stage of the study. The research has been also approved by the national engineering laboratory of point-of-care testing (Wondfo Biotech company, Guangzhou, China).

The diagnosis of malaria follows *Diagnostic Criteria for Malaria* issued by the Ministry of Health of the People's Republic of China. Patients were included if they ①came from relatively serious malaria epidemic area and ②suffered from fever of unknown origin. Patients were excluded if they ①suffered from vivax malaria or ②had mixed infections. The samples were confirmed as single malignant malaria infection by microscopic examination of thick blood smear.

An expert medical technologist collected approximately 5 mL of blood from adult subjects and 3 mL from minor subjects by venipuncture. Thick and thin blood films were prepared in duplicate using two drops of blood for each sample. The remaining blood was preserved at −20°C in EDTA tube. Thick blood films were stained with 10% Giemsa and microscopic examined by experienced technicians.

Positive references provided by WHO were divided into 5 geographical areas, comprising Africa (Benin I and Nigeria XII), the Americas (Santa Lucia), Australia (Papua New Guinea-FC27/A3) and Southeast Asia (Philippines- PH1).

### Expression and purification of HRP 2 protein

*Plasmodium falciparum* genomic DNA was extracted from clinical blood samples provided by Prof*.* Hui Liu (Yunnan Provincial Institute of Parasitic Diseases, Simao, Yunnan, China), using QIAamp DNA Mini Kit (QIAGEN, Germany). Pair of oligonucleotide primers was designed, complementary to forward and reverse strands of the HRP 2 exon II based on the sequence obtained from GenBank (U69551.1). The forward primer was HRP 2-F: 5׳-TATACATATGAATAATTCCGCATTTAATAATAACT-3׳, and the reverse primer HRP 2-R: 5׳-TACAGTCGACATAGACGTACTTCTTTTCGTAAG-3׳. The forward and reverse primers were designed to contain NdeI and XhoI restriction sites, respectively. Restriction endonucleases (*Nde*I and *XhoI*), DNA markers and PCR mix were purchased from Dongsheng Biotech Company (Guangzhou, China), while the T4 DNA Ligation mix was obtained from TaKaRa Biotech Co. Ltd (Dalian, China). The HRP 2 gene was cloned into a pET30a vector and transformed into CaCl_2_ competent *E. coli* BL21 (DE3) cells [17]. pET-30a plasmid and BL21 (DE3) cells were provided by Prof. Jufang Wang of South China University of Technology. Starter culture was grown overnight at 37°C in 5 ml LB medium, supplemented with kanamycin (50 mg/L). This stationary phase culture was used to inoculate 1 L LB medium to an OD600 of 0.6. The latter culture was inducted with 1 mM IPTG for target protein expression at 30°C and 225 g, overnight. Cells were harvested by centrifugation at 8000 g for 3 min and cell pellets resuspended in lysis buffer consisting of 100 mM Tris–HCl, pH 9.6. Cell suspensions were ultrasonicated on ice and centrifuged at 4°C at 12000 g for 15 min. The resulting supernatants were purified on a Hi-Trap Ni^2+^ column (GE, USA) using AKTA Purifier and the purified recombinant HRP 2 protein (rHRP 2) fractions visualized on 12% Sodium dodecyl sulfate-polyacrylamide gel electrophoresis (SDS-PAGE) [18]. The protein samples (>90% purity) were purified by dialyzing against 50 mM carbonate buffer, pH 9.6.

### Animal immunization and mAb production, screening, purification and characterization

Six-to-eight-weeks-old Balb/c mice were purchased from the Animal Laboratory Center of Sun Yat-sen University (Guangzhou, China). All experimental procedures were in compliance with the Guide for Care and Use of Laboratory Animals (NIH version, revised 1996). All animals were allowed free access to food and water.

MAbs were produced as described by Kohler and Milstein [19] and screened by indirect ELISA described below. The IgG fractions were prepared by ammonium sulfate precipitation and purified on Protein A column. The titers were determined by indirect ELISA and mAb isotyping was carried out using mouse mAb isotyping kit purchased from Sigma-Aldrich (St. Louis, MO, USA) according to the manufacturer’s instructions. The specificity of monoclonal antibodies was determined by their binding affinity to both rHRP 2 and *P. falciparum* infected blood samples. SDS-PAGE was carried out on 12% separating and 5% stacking gels, followed by protein transfer to polyvinylidene fluoride (PVDF) membranes. SuperSignal West Pico Chemiluminescent Substrate (Thermo Fisher Scientific Inc., USA) was applied for visualization. Determination of antibody affinity was carried out by a noncompetitive ELISA as described by David Beatty [20] with little modifications.

### Indirect ELISA

Microtiter plates were coated with 1 μg/ml of purified rHRP 2 in coating buffer (0.05 M carbonate buffer, pH 9.6). After overnight incubation at 4°C, plates were rinsed with washing solution (0.15 M PBS 0.1% Tween 20, pH 7.4) and blocked with 3% bovine serum albumen (BSA) for 2 h. 100 μL hybridoma cell supernatants were added to wells and incubated for 1 h at 37°C. After washing, 100 μL goat anti-mouse IgG-HRP (ZSGB-BIO, China, 1:20000) antibodies were dispensed into each well followed by 1 h incubation at 37°C. The enzymatic reaction was visualized using TMB substrate with hydrogen peroxide and stopped with 2 M Sulphuric acid. Optical density (OD) was measured spectrophotometrically at 450 nm. Samples were considered positive with OD of cell supernatant (P) to negative control (N) ratios greater than 2.1.

### Sandwich ELISA

Purified mAbs were paired, labeled with periodate oxidation and sandwich ELISA was performed as described before [21]. The lower the OD, the two strains of antibody antigen epitope closer; the higher the OD value, illustrate both the antigen epitope is more far.

### Establishment of POCT lateral immunochromatographic assay

HRP 2 specific mAb 1A5 (1.5 mg/ml) and goat-anti-mouse IgG (2.0 mg/ml) were diluted in 0.05 M carbonate buffer and sprayed on a nitrocellulose membrane at 1.5 and 1 μl/cm^2^ to form test and control lines, respectively. The prepared nitrocellulose membranes were kept in a dry pack at 30°C and 30% humidity. 1C10 antibody were conjugated to colloidal gold as described by Oliver [22] and sprayed on glass fiber at 12 μL/cm^2^. The strip was assembled by placing 0.7 cm of antibody-gold conjugate glass fiber a few centimeters from the test line and 4 mm wide test strips were cut. Test strip preparation was done in a good manufacturing practice (GMP) workshop.

### Sample evaluation and sensitivity calculation

Positive control samples provided by WHO were evaluated alongside 516 clinical samples and test results were compared with microscopic examinations. About 5 μL of each sample was applied to the sample site and three drops of dilution buffer added. Test strips were observed for 15 min and results were interpreted as positive when both the control and test lines turned red and negative if only the control line turned red. In cases where the control line did not appear, the results were considered invalid and the assay repeated.

Sensitivity of the test equals the ratio of total positives to total number of samples, specificity equals the ratio of total negatives to total number of samples, positive predictive value equals the proportion of the number of positive cases detected among actual positives cases and negative predictive value equals the proportion of the number of negative cases detected in relation to actual negatives.

### Statistics

Statistical analysis was performed with SPSS 18.0 software (SPSS, Chicago, IL). Data obtained from both microscopic examination and immunochromatographic test were analyzed. Sensitivity and specificity of the assay and how it compares with the standard microscopic examination was analyzed. Agreements between both data were computed by Kappa statistics. Differences were considered statistically significant at p < 0.05.

## Results

### Malaria diagnosis

Microscopic inspection showed that most samples were positive for only *Plasmodium falciparum* infections, with no mixed infections detected (n=322) including 60/90 (66.7%) samples from Henan, China, 105/202 (52.0%) samples from Yunnan, China and 157/224 (70.1%) from Myanmar. Negative Plasmodium falciparum blood samplesn=194) were also assessed by thick and thin smear microscopic examinations and confirmed to be *Plasmodium falciparum* negative, including 30/90 (33.3%) samples from Henan, China, 97/202 (48.0%) samples from Yunnan, China and 67/224 (29.9%) from Myanmar.

### mAbs 1A5/1C10 efficiently recognize recombinant and nature HRP 2

**Figure 2:**
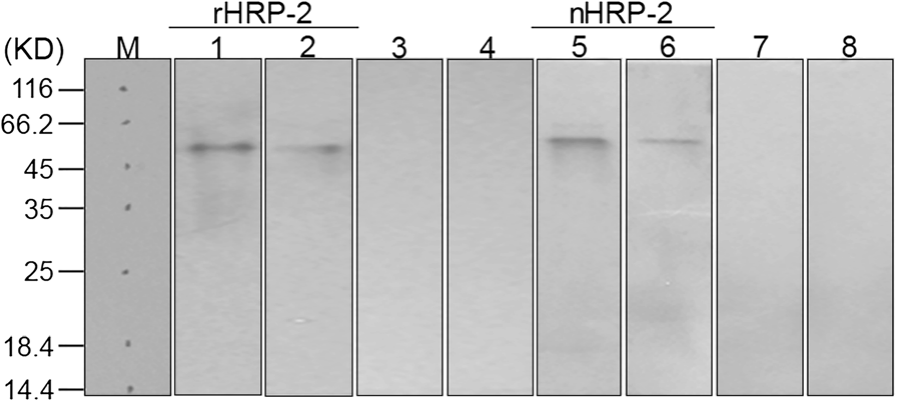
Western blot analysis of mAbs. Recombinant HRP 2 (0.5 μg) and nature HRP 2 were run on SDS-PAGE. The gel was blotted onto a nitrocellulose membrane and probed with 0.5 μg/ml of mAb 1A5 and 1C10. Lane M: molecular marker; Lane 1 and 2: rHRP 2 probed with mAb 1A5 and 1C10, respectively; Lane 3 and 7: Human blood serum probed with mAb 1A5 and 1C10, respectively; Lanes 5 and 6: Plasmodium falciparum cell culture supernatants probed with mAb 1A5 and 1C10, respectively; Lane 4 and 8: pLDH antigen (0.5 μg) probed with mAb 1A5 and 1C10, respectively.

The 831 bp DNA fragment was transformed into *E. coli* BL21 to express soluble recombinant HRP 2 exon II after IPTG induction. Hybridomas were developed using mouse spleen cells screened by indirect ELISA. After three rounds of limiting dilution, 5 stable cell lines producing mAb against PfHRP 2 were selected. mAbs from the 5 cell lines were paired in a sandwich ELISA (totally 10 pairs) and 1A5/1C10 pair was chosen for further analysis and detection of clinical samples. The titer values for mAb 1A5 and 1C10 were greater than 10^6^ and their affinity constants were 7.15 and 4.91 × 10^−7^ L/mol, respectively. Both mAbs were of the IgG1 isotype. Figure 2 shows the immunoblot analysis of mAbs 1A5 and 1C10 probing rHRP 2, human blood serum, *Plasmodium falciparum* cell culture supernatants and pLDH antigen. The result showed that mAbs 1A5 and 1C10 recognize recombinant and nature HRP 2 sensitively and spectively.

**Figure 3:**
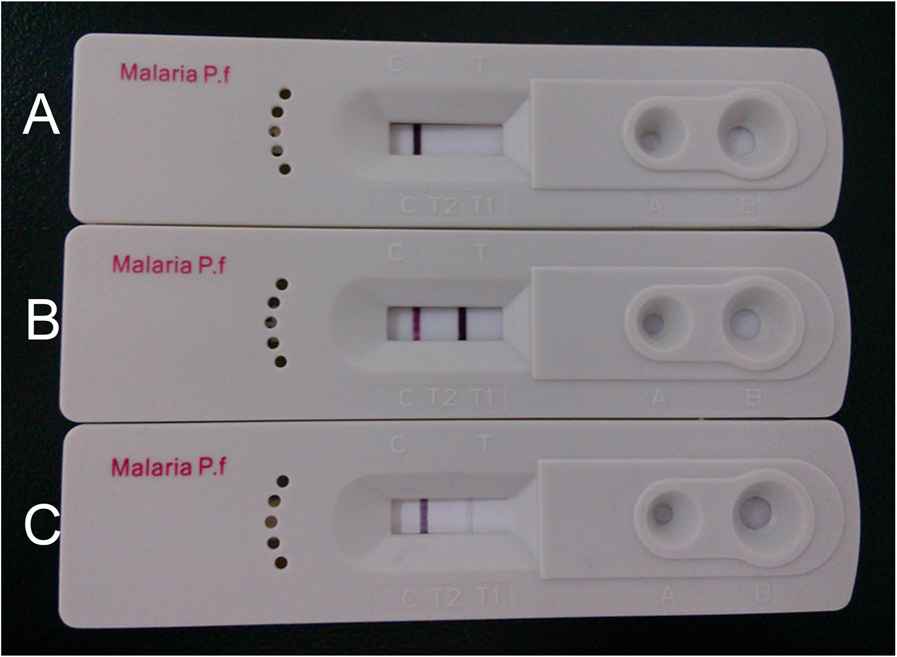
POCT lateral immunochromatographic assay. **A**, a representative of negative control in which only the control line turns red; **B**, a representative of positive control in which both the control and test lines turn red; **C**, a representative of sample in which both the control and test lines turn red and indicate that this sample is *P. falciparum* positive.

### Lateral immunochromatographic test strip evaluation and accuracy

All positive references were examined retrospectively with both microscopy and the immunochromatographic assay. Differences in detection levels were noted (Figure 3). The different positive references showed varied concentrations of HRP 2 antigen that resulted in significantly different detection levels (Table 1).

**Table 1:** **mAbs 1A5 and 1C10 double antibody sandwich assay detection of positive reference samples from WHO**

**Sample**	**HRP 2 Sequence information**	**Detection level**
Benin I	A	+
Santa Lucia	B(high)	+++
Nigeria XII	B(medium)	++
FC27/A3	B(low)	++
PH1	C	+

A, B and C represent different HRP 2 sequences.

“+” indicates weak positive, “+ +” indicates fairly positive and “+ + +” indicates strong positive.

### Screening of clinical samples

A total of 516 blood samples collected from 6–50 years old patients were examined. The newly developed test kit detected 164 of the 165 positive samples originating from China (sensitivity of 99.39%), with no false positives in the 127 negative samples examined. In samples collected in Myanmar area, a total of 157 positive and 67 negative cases were found, with a sensitivity rate of 98.72%. Three false negatives were recorded. These results are summarized in Table 2. Parasitemia density higher than 2000 and 200 parasites/μL for first time and recurrent cases, respectively, are evidence of higher infection rate. The POCT method was also used to screen clinical samples with parasites both below 200 and above 2000 parasites/μL in order to compare their sensitivity rate. Among the 16 clinical blood samples with less than 200 parasites/μL, two false negatives were obtained while only one false negative was recorded among the 77 microscopically positive samples with parasite density of 200–1999 parasites/μL (Table 3). There was 100% sensitivity for blood samples with higher densities above 2000 parasites/μL (Table 3).

**Table 2:** **Evaluation of immunochromatographic test in clinical sample screening**

**Evaluation criteria**	**China (n=292)**	**Myanmar (n=224)**	**Total (n=516)**
Sensitivity (95% CI)	99.39	98.72	99.07
Specificity (95% CI)	100	100	100
Positive predictive value (%)	100	100	100
Negative predictive value (%)	99.22	97.10	98.48

**Table 3:** **Comparison of immunochromatographic test strips and microscopic examination of thick blood smear**

**Parasite density**	**Sample size (n)**	**Positives**	**Detection rate**
<200	16	14	87.50%
200-1999	77	76	98.70%
>2000	229	229	100%

## Discussion

Malaria is a major public health problem worldwide and its total eradication is one of the urgent aims of the United Nations Millennium Development Goals. Hence the use of fast, accurate, and easy on-site detection methods and reagents as monitoring medical tools for early diagnosis and treatment of malaria in regions of high transmission and prevalence is particularly important. However, the performance of existing HRP 2 tests in areas of intense malaria transmission varies by age and prevalence of *P. falciparum* infection [14]. The particularly low specificity among children results in over-estimation of malaria infection prevalence in this group [23].

In the current study, we aimed to evaluate the immunogenicity of HRP 2 exon II for use in developing mAbs against HRP 2 for POCT. We have successfully expressed recombinant HRP 2 exon II in *E. coli* and purified its full amino acid sequence. After administration of the purified rHRP 2 antigen in mice followed by fusion of mouse splenocytes with myeloma cells, two different hybridomas (1A5 and 1C10) producing anti-HRP 2 mAbs were selected and used for the preparation of ascitic fluids in mice. After purification of ascitic fluids by ammonium sulfate precipitation and Protein A column, specificity of 1A5 and 1C10 was assessed by immunoblot: both antibodies exhibited remarkable specificity for detection of both recombinant and native *P. falciparum* HRP 2 antigen, binding to no other protein in human blood serum.

A noncompetitive ELISA method was used in the determination of antibody affinity. We found association constant (Ka) values of 7.15 x 10^−7^ and 4.91 × 10^−7^ (unit) for 1A5 and 1C10, respectively, an indication of the high binding affinity of these two mAbs. The high affinity observed for the expressed exon II region of PfHRP 2 suggests that epitopes are conserved in this region. Therefore, the recombinant PfHRP 2 protein developed in this study can serve as immunogen to facilitate the production of high affinity and high specificity mAbs for use in clinical diagnosis of malaria caused by *P. falciparum*. The genetic diversity of HRP 2 affects the performance of the HRP 2-based RDT [24].

Microscopic examination of both thick and thin blood smears is a traditional detection method and hence considered the gold standard for malaria diagnosis. However, due to its complicated operation and the requirement of experienced laboratory technicians, field application is limited. POCT, a new concept for rapid diagnosis of clinical samples developed in recent years is very crucial. POCT is based on small, portable, easy to operate, fast, accurate, and effective devices for on-site monitoring of infections. Sensitivity of HRP detection kits is affected by several factors including the quality of mAbs used, immunogenic response of B cells to the different epitopes of HRP 2 protein [25], and the polymorphism observed in the HRP gene sequence. The HRP antigen contains numerous repeats of histidine and alanine amino acids. The tripeptide repeat Ala-His-His in HRP 2 is quite conserved, while Ala-Ala-Asp which also has only slight variations can be detected by mAbs specific to HRP 2 in different isolates [26-28].

The lateral immunochromatographic assay was developed with the two HRP 2 specific mAbs and used for testing of blood samples collected from high endemic regions in China and Myanmar. There was a strong agreement between the immunochromatographic test results and microscopic examination among the 322 positive and 194 negative samples tested (K=0.9955 at p < 0.001). The observed sensitivity and specificity were 99.07 and 100%, respectively (Table 2). In clinical samples, there were false negatives at low parasite densities. However, samples with parasite densities above 2000 parasites/μL were detected with 100% sensitivity (Table 3).

mAbs with acceptable sensitivity, specificity and high affinity for the PfHRP 2 antigen are prerequisite for accurate diagnosis of *P. falciparum* infections. Because of the limited supply of mAbs, it is very likely that most HRP 2-based RDTs use similar or identical antibodies. Existing HRP 2-based RDTs are known to yield false-positive results [14]. Therefore, development of new mAbs with high sensitivity for use in HRP 2 diagnostic test kits is very important. We have evaluated two such mAbs and experiments are underway to further define the specific epitopes recognized by these mAbs and develop serological test kits using these mAbs. The newly designed RDTs combining two different mAbs would improve the detection of malaria worldwide given that the epitopes involved are common to all *P. falciparum*, resulting in saved lives and reduced occurrence of parasite resistance.

## Conclusion

The Plasmodium falciparum HRP 2 exon II contains immunogenic sites similar to those on the native antigen. These immunogenic sites can effectively be utilised for the development of highly sensitive and specific mAbs to facilitate malaria diagnosis in endemic communities.
